# Involvement of the Dorsal Vagal Complex in Alcohol-Related Behaviors

**DOI:** 10.3389/fnbeh.2022.801825

**Published:** 2022-03-07

**Authors:** Bailey N. Keller, Andras Hajnal, Kirsteen N. Browning, Amy C. Arnold, Yuval Silberman

**Affiliations:** Neural and Behavioral Sciences, Penn State College of Medicine, Hershey, PA, United States

**Keywords:** alcohol use disorder, gut-brain axis, interoception, nucleus of the tractus solitarius, vagus nerve

## Abstract

The neurobiological mechanisms that regulate the development and maintenance of alcohol use disorder (AUD) are complex and involve a wide variety of within and between systems neuroadaptations. While classic reward, preoccupation, and withdrawal neurocircuits have been heavily studied in terms of AUD, viable treatment targets from this established literature have not proven clinically effective as of yet. Therefore, examination of additional neurocircuitries not classically studied in the context of AUD may provide novel therapeutic targets. Recent studies demonstrate that various neuropeptides systems are important modulators of alcohol reward, seeking, and intake behaviors. This includes neurocircuitry within the dorsal vagal complex (DVC), which is involved in the control of the autonomic nervous system, control of intake of natural rewards like food, and acts as a relay of interoceptive sensory information via interactions of numerous gut-brain peptides and neurotransmitter systems with DVC projections to central and peripheral targets. DVC neuron subtypes produce a variety of neuropeptides and transmitters and project to target brain regions critical for reward such as the mesolimbic dopamine system as well as other limbic areas important for the negative reinforcing and aversive properties of alcohol withdrawal such as the extended amygdala. This suggests the DVC may play a role in the modulation of various aspects of AUD. This review summarizes the current literature on neurotransmitters and neuropeptides systems in the DVC (e.g., norepinephrine, glucagon-like peptide 1, neurotensin, cholecystokinin, thyrotropin-releasing hormone), and their potential relevance to alcohol-related behaviors in humans and rodent models for AUD research. A better understanding of the role of the DVC in modulating alcohol related behaviors may lead to the elucidation of novel therapeutic targets for drug development in AUD.

## Introduction

The dorsal vagal complex (DVC) plays a critical role in relaying interoceptive sensory information to higher order brain regions as well as mediating central output to abdominal viscera via the utilization of various neurotransmitter and neuropeptide systems (Rinaman, 2011; Browning et al., 2017; Browning and Carson, 2021). Many of these same neurotransmitters and neuropeptides have also been implicated in various aspects of alcohol use disorder (AUD) (Bodnar, 2013; Haass-Koffler et al., 2018; Jerlhag, 2018; Genders et al., 2020; Torruella-Suárez and McElligott, 2020; Ballaz et al., 2021). The DVC, therefore, may be involved in AUD through its various roles in interoceptive functions and signaling systems, but previous research has not fully investigated this hypothesis. Currently, there is a paucity of research directly linking alcohol-related behaviors and alteration in DVC function however, this review will demonstrate that there is substantial evidence which strongly suggests the potential role that the DVC may play in AUD.

**FIGURE 1:**
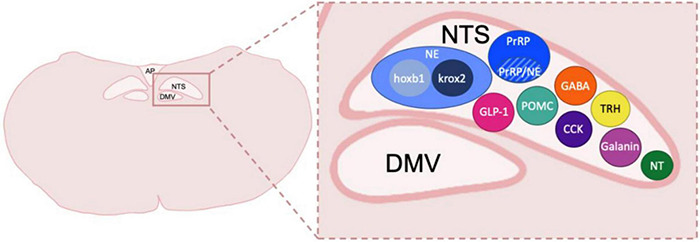
Neuronal types and subtypes in the NTS. Schematic coronal medullary section showing the diversity of cell types located within the NTS. Peptide and transmitter expression includes NE, norepinephrine, which is made up of cellular subtypes hoxb1 and krox2; prolactin-releasing peptide, PrRP which also has a population of neurons that co-express NE; GLP-1, glucagon-like peptide 1; POMC, pro-opiomelanocortin; GABA, gamma-aminobutyric acid; CCK, cholecystokinin; TRH, thyrotropin-releasing hormone; Galanin; NT, neurotensin. Other peptides shown to be in the DVC but not depicted include Met-enkephalin, Dynorphin, Substance P, calcitonin gene related peptide and Neuropeptide Y. NTS, nucleus of the tractus solitarius; DMV, dorsal motor nucleus of the vagus nerve; AP, area postrema. Sizes not to scale or indicative of typical location within a caudal DVC section.

The DVC, which contains the nucleus of the tractus solitarius (NTS), dorsal motor nucleus of the vagus (DMV) and area postrema (AP), is localized in the caudal dorsomedial portion of the medulla oblongata and is a crucial component of the autonomic nervous system (Rinaman, 2003b,^2011^; Browning et al., 2017; Herman, 2018; Browning and Carson, 2021; Martinez and Kline, 2021). The NTS receives gustatory and visceral information from trigeminal, facial glossopharyngeal, and vagus nerve afferents. Thus, the NTS serves as the initial processing site for many peripheral signals and relays them to various nuclei in the lower brainstem, the limbic system, and hypothalamus. The NTS also sends projections to the DMV, which predominately contains pre-ganglionic parasympathetic motoneurons that innervate the gastrointestinal, pulmonary, and cardiovascular systems and may be a critical locus of alcohol-related end organ dysfunction and peripheral neuropathy. Finally, the AP is a circumventricular organ which is highly vascularized and has access to circulating hormones and other factors such as toxins and cytokines outside of the central nervous system. These circulating chemical messages can be transformed by neurons in the AP to neural input that regulates DVC function. The AP also receives input from the vagus nerve and projects to multiple relay regions such as the NTS, locus coeruleus (LC), parabrachial nucleus (PBN), and ventral lateral medulla (Price et al., 2008). While the AP and the DMV are significant regions of the DVC, this review will primarily focus on NTS connections to corticolimbic and homeostatic areas in the forebrain previously linked to AUD development. Additionally, the NTS contains neuronal subtypes that produce various neurotransmitters and neuropeptides, systems that have previously been shown to be involved in alcohol-related behaviors (Figure 1). A better understanding of how the NTS and DVC in general modulate forebrain and peripheral pathways may be critical for potential new conceptual and therapeutic advancement for AUD and other substance use disorders.

## Nucleus of the Tractus Solitarius Afferents to Higher Order Brain Regions

**FIGURE 2:**
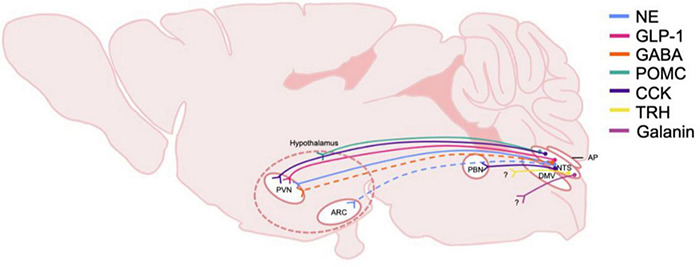
Schematic of DVC projections hypothesized to be involved in central homeostatic systems and AUD. The origin and distribution of central neurotransmitter and peptide pathways participating in consumption behaviors and energy homeostasis and potentially involved in AUD. Peptide and transmitter systems include NE, norepinephrine; GLP-1, glucagon-like peptide 1; GABA, gamma-aminobutyric acid; POMC, pro-opiomelanocortin; CCK, cholecystokinin; TRH, thyrotropin-releasing hormone; Galanin. These projections are derived from the NTS, nucleus of the tractus solitarius; and project to multiple nuclei in the hypothalamus including the PVN, paraventricular nucleus, and the ARC, arcuate. The general area delineating the hypothalamus is denoted with dashed lines. Note that the specific hypothalamic regions that NTS POMC neurons project to is not yet defined, thus nerve endings are shown to terminate in the general hypothalamic region. CCK neurons are shown to project to the PBN, parabrachial nucleus, which also projects heavily to the hypothalamus. Dashed lines indicate that projections have been identified, but functionality and relevance to AUD is not known. Question marks indicate that immunohistochemically, neurotransmitters or peptide are present however, the termination of the projections have not been identified. DMV, dorsal motor nucleus of the vagus nerve; AP, area postrema.

**FIGURE 3:**
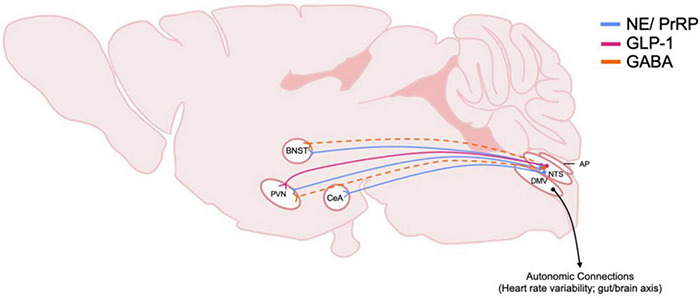
Schematic of DVC projections hypothesized to be involved in central stress systems and AUD. The origin and distribution of DVC neurotransmitter and peptide pathways involved in stress and implicated in AUD. Peptide and transmitter systems include NE, norepinephrine; PrRP, prolactin releasing peptide; GLP-1, glucagon-like peptide 1; GABA, gamma-aminobutyric acid. Dotted lines indicate that projections have been identified, but functionality and relevance to AUD is not known. Note that most of these projections arise from the caudal NTS, nucleus of the tractus solitarius. Also, efferent motor projections from the DMV, dorsal motor nucleus of the vagus nerve, are included to highlight their importance in physiological responses to stress in AUD. BNST, Bed nucleus of the stria terminalis; PVN, paraventricular nucleus of the hypothalamus; CeA, central amygdala; AP, area postrema.

The NTS regulates many behavioral responses to internal and environmental stimuli via its projections to forebrain regions. The NTS has been shown to target circuits involved in control of energy homeostasis such as the paraventricular nucleus of the hypothalamus (PVN) (Figure 2; Rinaman, 2010). Studies utilizing anterograde tracers microinjected into the caudal NTS as well as glucagon-like peptide 1 (GLP-1) positive immunological staining demonstrate the ascending pathways of these neurons (Rinaman, 1999). The NTS sends direct, stress-sensitive projections to both corticotropin releasing hormone (CRH) neurons and oxytocin neurons within the PVN that regulate hypothalamus-pituitary-adrenal (HPA) axis mediated stress responses (Figure 3). Finally, while not a focus of this review, the NTS projects to brainstem areas involved in control of cardiovascular parasympathetic and sympathetic functions including the nucleus ambiguous and the caudal and rostral ventrolateral medulla, respectively.

**FIGURE 4:**
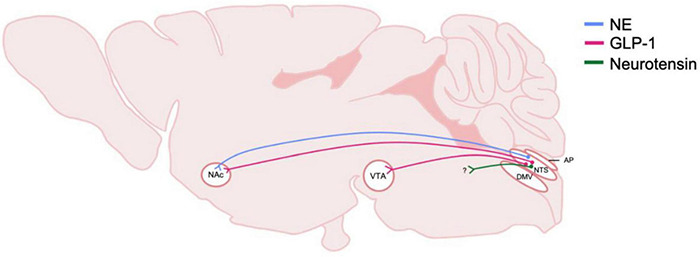
Schematic of DVC projections hypothesized to be involved in central reward systems and AUD. The origin and distribution of central neurotransmitter and peptide pathways hypothesized to be involved in rewarding properties of alcohol. Peptide and transmitter systems include NE, norepinephrine and GLP-1, glucagon-like peptide 1 and neruotensin. These projections are derived from the NTS, nucleus of the tractus solitarius; and project to mesolimbic regions including the NAc, nucleus accumbens, and the VTA, ventral tegmental area. Question marks indicate that immunohistochemically, neurotransmitters or peptide are present, however, the termination of the projections have not been identified. DMV, dorsal motor nucleus of the vagus nerve; AP, area postrema.

The NTS also projects to the ventral tegmental area (VTA) and nucleus accumbens (NAc), areas involved in the rewarding properties of food and alcohol as well as other drugs (Figure 4; Alhadeff et al., 2012). The NTS also has direct projections to regions such as the prefrontal cortex, central amygdala (CeA), and bed nucleus of the stria terminalis (BNST) which suggests that the NTS has a role in processing cognitive and emotional responses to stress (Schwaber et al., 1982) as well in negative reinforcing properties of alcohol and other substances (Harris and Aston-Jones, 2007; Smith and Aston-Jones, 2008; Koob and Volkow, 2016). In addition to homeostatic stressors, the NTS is sensitive to other stressors including restraint stress, forced swim and immobilization stress, which all have been shown to increase *c-fos* expression (Pezzone et al., 1993; Cullinan et al., 1995). The NTS is also sensitive to chronic variable stress as demonstrated by increased delta FosB immunoreactivity (Flak et al., 2012).

Overall, projections to such higher order regions positions the NTS and the visceral sensory information it relays as a mediator of the rewarding properties of food and alcohol as well as stress sensitivity and negative reinforcement. However, the role that NTS projections play in regulating AUD or substance use disorder development remains to be fully elucidated (Luckman and Lawrence, 2003). Below, we will discuss the roles that multiple neurotransmitters and neuropeptides produced by the DVC play in the modulation of intake and reward behaviors and their possible relevance to alcohol intake behaviors.

## Neurotransmitters

### Norepinephrine

Norepinephrine (NE) is a catecholamine transmitter that is derived from hindbrain nuclei that have projections throughout the brain. It is synthesized in neurons that contain the enzyme dopamine-β-hydroxylase (DβH) which modifies dopamine into NE (Dahlström and Fuxe, 1964). The most well studied source of NE in terms of behavioral functions is the LC. Noradrenergic neurons in the LC play a critical role in regulating behavioral processes under stress conditions as well as cognitive functions including motivation, attention, learning, and memory (Schwarz and Luo, 2015). NE derived from the NTS has classically been examined as a significant signaling component of the sympathetic nervous system, and is involved in life sustaining physiological functions such as cardiovascular regulation and glucose homeostasis. However, NTS-derived NE projections are also intertwined in reward and motivational circuits such as those found in the lateral hypothalamus, VTA, NAc, and extended amygdala (Rinaman, 2011). Additional evidence shows the LC receives input from NTS neurons and may help to modulate NE signaling in the cortex, hippocampus, amygdala, thalamus, and cerebellum indirectly through modulation of LC projections to these areas (Rinaman, 2010; Fox et al., 2016). For the scope of this review, we will focus on NE projections arising from the NTS and their involvement in alcohol related behaviors. Noradrenergic cell bodies in the NTS, also called the A2 nucleus, ascend their processes through the ventral noradrenergic bundle (VNAB). These connections from A2 neurons allow for relaying and processing of interoceptive information to cortical and subcortical brain regions which then influence homeostatic and cognitive functioning as well as affective states. The NTS system also has a separate genetic lineage from NE neurons in the LC (Robertson et al., 2013) that may have opposing roles in regulating anxiety-like behaviors. Central NE systems, which are crucial for the stress response, are highly conserved across mammalian species; therefore, understanding this system in preclinical models has significant clinical relevance.

NE modulates neurotransmission pre- and post-synaptically through G-protein coupled adrenergic receptors (ARs) (Strader et al., 1995). In addition to residing in cortical and subcortical brain regions, ARs play a major role within the peripheral nervous system especially in terms of cardiovascular functioning (Reid, 1986; Philipp et al., 2002; Chen and Minneman, 2005). These receptors belong to either α1, α2, or β-ARs families and are modulated by endogenous epinephrine or norepinephrine (Rohrer and Kobilka, 1998). α1-AR are typically G_*q*_ coupled; however it has been reported that α1-AR are sometimes coupled to G_*i*_ and G_*o*_ proteins (Nestler et al., 1996). α2-AR are G_*i*_ coupled and hypothesized to be primarily autoreceptors on presynaptic NE terminals to regulate NE signaling; however some studies indicate a heterosynaptic role of α2-AR, particularly in the extended amygdala (Snyder and Silberman, 2021). Lastly, β-ARs typically utilize G_*s*_ signaling and are particularly important in stress/AUD interactions (Gilpin and Koob, 2010; Haass-Koffler et al., 2018; Snyder and Silberman, 2021). Postsynaptic regulation of target circuits by NE varies depending on the brain region and receptor expression. NE neurons in the NTS also have been suggested to co-release other transmitters and peptides such as glutamate (Stornetta et al., 2002), neuropeptide Y (Sawchenko et al., 1985), dynorphin (Ceccatelli et al., 1992), nesfatin-1 (Bonnet et al., 2009), neurotensin (Riche et al., 1990), pituitary adenylate cyclase-activating polypeptide (Das et al., 2007), galanin (Melander et al., 1986), and prolactin-releasing peptide (PrRP) (Chen et al., 1999). In the following sections, we will discuss NTS NE systems and their roles in feeding, reward, motivation, and the stress response, and how these systems may be recruited and modified in AUD.

#### Nucleus of the Tractus Solitarius Norepinephrine Involvement in Stress and Anxiety-Related Behaviors

The ascending NTS NE system may have a significant role in modulating anxiety-like behaviors. NE has strong regulatory control over limbic circuits, which includes projections from the NTS to the BNST and CeA, regions both associated with modulating behavioral responses to stressful stimuli (Phelix et al., 1992; Park et al., 2009). NTS projections to the limbic brain may be activators of aversive emotional states such as anxiety, fear, and depressive-like behaviors (Itoi and Sugimoto, 2010). These neurons are activated in response to stressful stimuli such as noxious interoceptive stimuli and immune challenge (Gaykema et al., 2007), as well as salient stressful stimuli such as predator odor exposure (Myers and Rinaman, 2005). There are dense projections from NTS NE neurons to the BNST, a functionally heterogeneous region of the extended amygdala implicated in the modification of physiological and behavioral responses to stress (Phelix et al., 1992, 1994; Terenzi and Ingram, 1995; Aston-Jones et al., 1999). LC NE projections to the BNST also exist, however they are much less dense in comparison (Fox et al., 2016). Additionally, the NTS accounts for 5% of NE projections to the CeA (Petrov et al., 1993; Davis et al., 1994; Khoshbouei et al., 2002).

NTS NE neurons selectively target the medial parvocellular (mp) neurons in the PVN (Sawchenko and Swanson, 1981, 1982; Cunningham and Sawchenko, 1988). This brain region contains CRH neurons, which organize and initiate the HPA axis stress response cascade. CRH neurons in the PVN receive direct synaptic input and regulation from NTS NE neurons as shown by light and electron microscope analysis (Liposits et al., 1986a,b). Stressful stimuli have been shown to trigger NE release from NTS neurons thereby recruiting the HPA axis which then increases excitability of CRH neurons (Szafarczyk et al., 1985; Alonso et al., 1986; Rivier and Plotsky, 1986; al-Damluji, 1988; Plotsky et al., 1989; Pacak et al., 1995; Onaka et al., 1996; Onaka, 2004; Wittmann, 2008). These CRH neurons then produce and release CRH in response to excitation from NE neurons. Following CRH secretion, adrenocorticotropin hormone (ACTH) is released from the anterior pituitary into circulation where it activates cells within the adrenal cortex resulting in the synthesis and release of glucocorticoids. These glucocorticoids then provide a negative feedback inhibition on PVN neurons (Herman et al., 2016). NTS NE neurons also innervate PVN oxytocin neurons, which then inhibit PVN CRH neuron activity, potentially as a mechanism to regulate the gain of NE modulation of overall HPA axis function (Smith et al., 2016; Jamieson et al., 2017; Winter and Jurek, 2019). PVN oxytocin neurons reciprocally innervate the DVC as well (Llewellyn-Smith et al., 2012). Oxytocin projections to the NTS have also been shown to have important implications for stress adaptation and may be crucial for GI functionality in response to stress (Jiang et al., 2018), however there does not appear to be oxytocin producing neurons in the DVC.

The NTS NE projections to the hypothalamus appear to be functionally malleable. Altering NE input to mpPVN neurons through lesions or other methods leads to attenuated CRH neuronal responses to interoceptive signaling (Ritter et al., 2003; Bienkowski and Rinaman, 2008). Lesions to the VNAB which affect NTS afferent projections to the PVN result in reductions in NE in the hypothalamus and thus reduced PVN CRH release (Alonso et al., 1986) and subsequent corticosterone release in response to stress (Gaillet et al., 1993). Additionally, NTS NE neurons are sensitive to modulation by glucocorticoids, making these circuits responsive to physiological and behavioral changes during acute and chronic stress (Herman and Cullinan, 1997). Therefore, NE release from NTS neurons into the PVN of the hypothalamus is critical for modulation of the stress response.

Modulation of the PVN by NTS NE seems to be specific to responsiveness of the HPA axis to certain types of stress. This is demonstrated in several studies where selective ablation of DβH-positive projections using saporin toxin impaired responses to stressors such as immune challenge (Li et al., 1996; Bienkowski and Rinaman, 2008), deprivation of glucose (Ritter et al., 2003), but not forced swim stress (Ritter et al., 2003). Microdialysis studies show that NE release in the PVN can occur from various acute stressors as well, where the amount of NE release in the PVN correlates to the amount of ACTH released into circulation (Pacák et al., 1995). Lesions within the VNAB decrease ACTH release and CRH expression in the PVN in response to a foot shock stressor in Sprague Dawley rats (Blandino et al., 2013). Even with the decrease in ACTH release, there was not a subsequent decrease in corticosterone responses (Blandino et al., 2013).

Other studies suggest that NE’s role in PVN activation may be more complicated. Chronic stress leads to HPA axis sensitization to signaling from NE inputs (Pardon et al., 2003) as well as increases in the amount of NE inputs to CRH neurons leading to increased HPA axis signaling (Flak et al., 2009). However, it is not known whether increased NE inputs are directly from NTS NE neurons. NE inhibits a population of parvocellular neurons in the PVN via a β-AR mechanism in *ex vivo* slices (Daftary et al., 2000; Han et al., 2002). Also, higher doses of infused NE can reduce release of CRH (Plotsky, 1987) potentially via oxytocin interactions as described above. This evidence suggests that NE’s influence on CRH release from the PVN may depend on the magnitude of NTS activation and which subcircuits are impacted within the PVN. NE release in the PVN may also affect local glutamatergic and GABAergic transmission. In *ex vivo* PVN slices, the excitatory effects of NE on parvocellular PVN neurons could be blocked by glutamate receptor antagonists and tetrodotoxin. This is indicative of NE regulating local glutamate release (Daftary et al., 1998). It is possible that NTS NE neurons co-release glutamate and NE in the PVN due to NTS NE neurons having glutamate transporters (Zhao et al., 2007). NE could also stimulate or inhibit GABAergic neuron activity in parvocellular neurons (Han et al., 2002). Thus, NTS NE possibly influences HPA activity through modulation of local circuitry in the PVN as well as via direct modulation of PVN CRH and oxytocin neurons.

#### Nucleus of the Tractus Solitarius Norepinephrine in Alcohol and Other Substance Use Disorders

NE transmission is highly responsive to alcohol and is critical for both positive and negative alcohol reinforcement (Koob and Volkow, 2016). In clinical studies, alcohol dependent individuals appear to have higher levels of NE metabolites in cerebral spinal fluid compared to matched controls after acute alcohol administration (Borg et al., 1981). This is also modeled in preclinical studies where there are higher levels of NE metabolites during intoxication and withdrawal in dependent animals that are administered alcohol daily via oral gavage (Karoum et al., 1976). Chronic alcohol administration via IP injection in adult male mice also sensitizes NE terminals to release more NE (Lanteri et al., 2008). These findings are indicative of increased NE transmission resulting from chronic alcohol use and dependency, but whether this increased NE transmission is dependent upon functional alterations in NTS NE neurons is not fully established.

So far, studies have shown an increase of c-Fos (a marker of neuronal activity) in tyrosine hydroxylase (TH) positive neurons in the NTS after IP or intragastric administration of alcohol (Thiele et al., 2000; Lee et al., 2011; Aimino et al., 2018). In the NTS in adult male mice, acute alcohol exposure to *ex vivo* brain slices via bath application enhances GABAergic signaling, likely onto other local circuit GABA neurons, resulting in disinhibition and increased c-Fos expression of NE neurons in the NTS (Aimino et al., 2018). NTS NE neurons have also been shown to be critical for conditioned place preference (CPP) for morphine in mice (Olson et al., 2006). Olson et al. (2006), demonstrated that deficits in CPP in mice with global genetic deletions DβH could be rescued when NTS NE was restored through viral reintroduction of DβH expression. Recovery of DβH expression in the LC however, did not rescue morphine-related CPP behaviors. This strongly suggests that NTS NE may be a common factor in the development of use disorders for numerous substances.

As discussed above, NTS NE projections to the PVN play a strong role in regulating HPA axis function (Herman and Cullinan, 1997). Chronic alcohol consumption is well known to be associated with disruption of the HPA axis and stress response (Koob, 2009; Stephens and Wand, 2012; Koob and Volkow, 2016; Sinha, 2018). A combination of activating HPA and stress circuits with aberrant NTS NE transmission and chronic alcohol exposure may create a vicious feed-forward cycle that exacerbates NE dysfunction (Retson et al., 2015). These alterations create allostatic changes in NE transmission following chronic alcohol exposure and withdrawal. The NTS also sends dense projections to the BNST, where NE is thought to be a critical signal in stress-induced reinstatement to drug seeking behaviors through modulation of local CRH neurons (Silberman and Winder, 2013; Snyder and Silberman, 2021). Interestingly, lesions of VNAB attenuates stress-induced reinstatement of opiates while lesions to LC NE neurons have no effect (Wang et al., 2001), highlighting the importance of NTS NE neurons specifically being involved in the aversiveness of withdrawal and stress-induced reinstatement. Therefore, NTS NE is suggested to be a main driver of the negative affective state that occurs during withdrawal and may be critical in reinstatement to drug seeking behaviors (Koob and Volkow, 2016).

#### Norepinephrine in Other Consumption Behaviors

NTS NE neurons are involved in suppressing and triggering feeding behaviors in various ways via bidirectional interactions with the hypothalamus. The hypothalamus has regulatory control over the initiation and termination of food intake through projections to caudal brainstem regions, including the NTS (Berthoud, 2002; Woods and D’Alessio, 2008). In turn, NTS projections to the hypothalamus modulate hypothalamic activity to regulate feeding (Ritter et al., 1975; Myers and McCaleb, 1980; Date et al., 2006; Rinaman, 2007). The NTS responds to gut-brain peptides such as ghrelin, which results in the release of NE into the arcuate nucleus of the hypothalamus (Date et al., 2006), a hypothalamic region known to play a critical role in the orexigenic properties of peripheral ghrelin signaling. In one study, selective ablation of NTS NE neurons resulted in elimination of ghrelin-induced consumption (Date et al., 2006). Complementary studies have shown that IP injections of ghrelin did not stimulate feeding in mice with vagotomies (Asakawa et al., 2001). Thus, NTS-mediated NE release into the hypothalamus is important for consumption behaviors regulated by peripheral ghrelin. This system may be targeted to help regulate alcohol consumption since clinical studies with alcohol-dependent individuals have shown that exogenous ghrelin administration increases alcohol craving and self-administration (Leggio et al., 2014; Farokhnia et al., 2018; Deschaine et al., 2021). NTS NE neurons are also important for signaling of anorexic gut-brain peptides. Selective ablation of NTS NE neurons attenuates hypothalamic activation by cholecystokinin (CCK) while CCK-induced anorexia was also attenuated (Rinaman, 2003a). Overall, selectively modulating NTS NE may be a way to influence sensitivity to endogenous and exogenous gut-brain peptides and may be consequential to intake behaviors for food and alcohol.

#### Additional Considerations of Nucleus of the Tractus Solitarius Norepinephrine Neuron Subtypes

A growing literature indicates that NTS NE neurons arise from distinct subtypes based on developmental origin and expression of distinct transcription factor genes. One of these neuronal subtypes express Krox20 while the other express Hoxb-1 (Figure 1; Robertson et al., 2013). These subtypes appear to be non-overlapping with activation resulting in distinct behavioral output. NTS Hoxb-1 neurons project directly to the insular cortex (Robertson et al., 2013; Chen et al., 2019). It can be inferred these inputs may serve to relay interoceptive signals directly to cortical regions to modulate arousal, affective states, and stress related behaviors (Gogolla, 2017; Chen et al., 2019). While the functional consequences of these projections have not been investigated in the context of alcohol-related behaviors, studies have shown that selective activation of Hoxb-1 neurons using DREADDs reduces anxiety-like behaviors in the context of acute stress through projections to the BNST (Chen et al., 2019). These results are in direct contrast to the known anxiogenic functionality of LC NE neurons (Robertson et al., 2013) and other known NTS NE neuron functions described in previous sections. This is one example of how examining cell type specific differences in neuronal physiology through recently developed genetic manipulating technology could reveal the heterogeneity of neuronal populations beyond their anatomical localization and should be further examined in the context of AUD.

## Gabaergic Projections

In previous studies, GABAergic neurons in the NTS were suggested to be local interneuron or neurons that projected to other adjacent brainstem regions such as the pons, medulla, and spinal targets such as the spinal nucleus of the trigeminal, parvicellular reticular nucleus, and the dorsal column nuclei (Kawai and Senba, 1999; Shi et al., 2021). Utilizing track tracing methods, a recent study demonstrated that NTS GABAergic neurons are also able to project to forebrain areas including the BNST and the PVN (Shi et al., 2021). Identifying these projections from the NTS to the BNST and PVN further suggests the NTS and interoceptive sensory information has a hand in regulating energy homeostasis, the stress response, and affective states of the brain. Given the well characterized interaction between alcohol and the GABAergic system in general, and our initial findings showing a potential for alcohol modulation of NTS GABAergic circuit function (Aimino et al., 2018), further examination of NTS GABA signaling may be a critical for the overall understanding of AUD development, but the functionality of these neurons needs to be further explored.

## Neuropeptides

Neuropeptides are short biomolecules ranging from 3 to 40 amino acids which are utilized for neuroendocrine and synaptic communication to regulate physiological functioning. The ubiquitous use of neuropeptides by various organ systems across different species highlights their significance in biological functioning. This section will review the extensive diversity of neuropeptidergic neurons that reside in the DVC and their potential relevance in the various aspects of AUD.

## Glucagon-Like Peptide 1

Glucagon-like peptide 1 (GLP-1) is a gut-brain peptide produced by enteroendocrine L-cells and neurons in the NTS that decreases food intake, regulates glucose homeostasis by stimulating insulin secretion, inhibits gastric emptying, and modulates glucagon secretion (Novak et al., 1987; Jin et al., 1988). Peripheral GLP-1 release into circulation is dependent upon taste receptor activation, ingestion, and possibly other hormonal signals such as CCK and gastric inhibitory peptide (Rocca and Brubaker, 1999; Hansen and Holst, 2002). While GLP-1 can cross the blood-brain barrier, it is degraded rapidly in circulation with a half-life of around 2 min (Herrmann et al., 1995). The NTS produces the most GLP-1 projections in the brain and is responsible for activating GLP-1 receptors (GLP-1R) centrally (Larsen et al., 1997; Merchenthaler et al., 1999). Since the half-life of and diffusion of circulating GLP-1 in the brain is limited, centrally produced GLP-1 is most likely mediating effects on brain regions non-adjacent to circumventricular organs (Kastin et al., 2002).

Immunological preparations outlining the anatomy of the DVC reveal that GLP-1 does not co-localize with catecholamines (Larsen et al., 1997). GLP-1 neurons have vesicular glutamate transporters suggesting that these neurons may corelease glutamate (Zheng et al., 2015). GLP-1R are conjugated with G_*s*_ or G_*i*_ proteins and activation of GLP-1R is typically associated with increased GABAergic activity or suppression of glutamatergic activity, such as the activity of GLP-1R found on presynaptic glutamatergic terminals in the cortex and hippocampus (Rebosio et al., 2018; Jerlhag, 2020). Since GLP-1R agonists are already approved for the treatment of type II diabetes and obesity due to its effectiveness in reducing food reward and craving, GLP-1 has become an attractive candidate for the treatment of substance use disorders including AUD (van Bloemendaal et al., 2014). Further mechanistic studies on the role of the GLP-1 system in AUD are needed.

### GLP-1 Effects on Reward Circuits Mediating Intake of Food and Alcohol

GLP-1R associated with reward are located in brain regions such as the VTA, NAc, the lateral septum and the paraventricular thalamus (PVT) which may be involved in GLP-1’s effects on alcohol-related behaviors (Dossat et al., 2011; Alhadeff et al., 2012; Dickson et al., 2012; Ong et al., 2017). Previous studies demonstrate that administration of GLP-1 results in decreased food intake through centrally mediated GLP-1 signaling (Kanoski et al., 2011), with previous studies focusing on homeostatic centers such as the PVN (Vrang et al., 2007). These studies demonstrate that central GLP-1 release is able to modulate food intake, however, other evidence suggests that central GLP-1 may act as a secondary circuit to induce satiation recruited by stress or large quantities of intake (Holt et al., 2019). More recent studies now point to GLP-1 mediated activity in the mesolimbic reward system as a component in regulating intake of natural rewards and drugs. Direct infusion of GLP-1R agonists into the VTA and NAc result in decreased intake of palatable rewarding food such as sucrose and high-fat diet, regular chow intake, and body weight in rats that were food-deprived overnight (Alhadeff et al., 2012). This indicates that GLP-1 signaling in the reward system decreases the motivation to feed, especially on highly palatable nutrients possibly through a mechanism of modulating dopamine signaling in VTA and the NAc. This is supported by other neuronal tracing studies showing that GLP-1 producing neurons in the NTS project directly to the brain regions involved in the reward system such as the VTA and the NAc (Alhadeff et al., 2012).

Preclinical studies found that the GLP-1R agonist AC3174 significantly reduced voluntary alcohol consumption in adult male mice. In this study, alcohol intake was assessed using the two-bottle choice paradigm where mice are given the option of drinking either water or alcohol. Voluntary drinking behaviors were measured before and after chronic intermittent ethanol exposure (CIE) where mice were exposed to ethanol vapors for 16 h a day for 4 consecutive days a week. Importantly, the decrease in alcohol consumption continued even after ceasing treatment with the GLP-1R agonist (Suchankova et al., 2015). In another preclinical study, animals treated with a Dipeptidyl-peptidase IV (DPP-IV) inhibitor, which inhibits the enzyme responsible for metabolizing GLP-1, demonstrate a delayed tolerance to alcohol’s anxiolytic effects and show decreased anxiety after alcohol withdrawal (Sharma et al., 2015). Since increased anxiety during alcohol withdrawal is a common aspect of relapse, these data suggest that targeting the GLP-1 system may decrease the chances of relapse. While GLP-1R agonists have consistently shown reduction in alcohol intake behaviors, the potential mechanisms involved are not always clear (Marty et al., 2020). The effects of altering endogenous GLP-1 and examining alcohol-mediated behaviors could reveal more insights about how GLP-1 could be used as a therapy. Additional studies are also needed to narrow down the effects of GLP-1R activation to differentiate between effects of peripheral activation versus central activation in attenuating alcohol-directed behaviors.

Highlighting the importance of central GLP-1R in the regulation of alcohol consumption, agonists of GLP-1R reduced alcohol-mediated behaviors in the two-bottle choice paradigm but such effects are lost when GLP-1R are selectively knocked out in the central nervous system. More specifically, GLP-1R activation has been shown to be involved in decreasing alcohol intake behaviors most likely due to the presence of GLP-1R in the mesolimbic reward system (Egecioglu et al., 2013; Shirazi et al., 2013; Suchankova et al., 2015; Vallöf et al., 2019; Marty et al., 2020). However, GLP-1R expression in other brain regions, such as the extended amygdala and PVN (Ghosal et al., 2013), may also play a role in reduction of alcohol intake in preclinical models via modulation of stress-reinstatement pathways, although this has not been directly tested and findings on GLP-1R activation on anxiety-like behaviors have been mixed. Additional evidence suggests that GLP-1R agonist therapies may also be effective in reducing opioid intake and seeking in preclinical models (Douton et al., 2021a,b). Overall, NTS GLP-1 neuron function in the context of AUD and stress could be a line of research parallel to the effects of NTS NE and stress on alcohol mediated behaviors.

Based on promising preclinical studies and relative safety of repurposing an already FDA-approved treatment for a novel indication, a number of clinical studies are underway examining the potential ability of GLP-1R agonists to reduce alcohol intake (Antonsen et al., 2018). While GLP-1 and AUD associations are not well established in human clinical populations, currently published research shows a number of promising findings. One study utilized a *post hoc* analysis on a case-control study found that GLP-1R polymorphisms were associated with AUD in humans and were related to increased intravenous administrations of alcohol as well as increased activation of the globus pallidus during rewarding feedback on a task that measures reward in an fMRI study (Suchankova et al., 2015). While initial clinical findings are generally promising, given the various roles that GLP-1 plays in consumption, reward, stress, and metabolic function, the effects of GLP-1 on multiple systems needs to be accounted for when interpreting effects on alcohol-related behaviors and as a potential therapy for AUD.

## Prolactin-Releasing Peptide

Prolactin-releasing peptide (PrRP) is classically known to stimulate prolactin release as the name suggests (Matsumoto et al., 1999). The canonical role of prolactin release is to serve as a nursing stimulus, however, it also influences reproductive behaviors, cognition, homeostasis, and immunological responses (Freeman et al., 2000; Cabrera-Reyes et al., 2017). PrRP functions in roles beyond mediating prolactin release, such as utilizing interoceptive information about the metabolic state of the organism to modulate the behavioral and endocrine stress response (Rinaman, 2007). There are PrRP neurons densely located in the NTS where PrRP is expressed in roughly 80% of catecholaminergic neurons (Maruyama et al., 2001; Maniscalco et al., 2012). Studies also show that PrRP neurons innervate magnocellular and parvocellular neurosecretory cells in the PVN (Matsumoto et al., 2000). There are PrRP receptors that also populate the PVN and participate in the stress-response as local administration of PrRP into the PVN increases ACTH (Roland et al., 1999; Matsumoto et al., 2000). Other studies found that coadministration of subthreshold NE and PrRP doses resulted in increased plasma ACTH, suggesting a critical interaction between PrRP and NE (Maruyama et al., 2001).

NTS PrRP neurons are sensitive to various stressful stimuli as shown by increased c-Fos expression in these neurons following acute restraint stress (Maniscalco et al., 2015), water immersion restraint stress (Maruyama et al., 2001), hemorrhagic stress (Uchida et al., 2010), and acute inflammatory stress (Mera et al., 2006). Chronic stress via repeated restraint was also shown to upregulate PrRP transcription in the NTS (Tóth et al., 2008). The activation of these neurons in response to stress also seems to be modulated through altered metabolic cues. For example, c-Fos expression following stress exposure is decreased in rats that are fasted (Maniscalco et al., 2015; Maniscalco and Rinaman, 2017). This implies that PrRP/NE neurons modulate acute and chronic stress-responses accordingly with consideration to metabolic state. Overall, these data suggest that PrRP release is relevant for stress modulation where PrRP and NE release may both synergistically fine tune HPA axis activity.

Thus far, how PrRP release into target areas such as the PVN affects alcohol-mediated behaviors has not been explored in depth. The close relationship between alcohol behaviors and stress circuitry, however positions PrRP as potential mediator of both. Thus, targeting the NTS PrRP system in conjunction with the NE system in AUD may lead to better outcomes when treating the negative withdrawal symptoms of AUD associated with anxiety and when addressing stress-induced relapse.

## Proopiomelanocortin

The melanocortin system is critical for control of energy homeostasis through its sensitivity to circulating nutritional stimuli and neuromodulatory signals. Proopiomelanocortin (POMC) is a precursor to multiple endogenous peptides such as α-melanocyte stimulating hormone (α-MSH), β-MSH, γ-MSH, β-endorphin, and ACTH (Harno et al., 2018). POMC neurons in the arcuate nucleus of the hypothalamus have been a heavy focus of study in a number of fields related to intake behaviors, but POMC neurons are also present in the NTS and their roles in intake behaviors is less well characterized (Joseph et al., 1983; Heisler and Lam, 2017). POMC expression in NTS neurons shows a lack of co-localization with cells that produce CCK, GLP-1, TH, or choline acetyltransferase (ChAT) which demonstrates that they are a distinct population of cells in the NTS (Georgescu et al., 2020). The NTS processes various metabolic cues from neuronal activity to circulating factors which position NTS POMC neurons to influence energy homeostasis. One study demonstrated how NTS POMC neuronal activity curbs food consumption acutely and chronically utilizing chemogenetics which reveals that NTS POMC neurons regulate feeding and energy homeostasis by incorporating peripheral adiposity signals with neurotransmission from the vagus signifying satiation (Zhan et al., 2013).

Alcohol alters signaling of endogenous opioid peptide systems including the POMC system. A 2-week administration of alcohol was shown to increase POMC mRNA expression in the hypothalamus, with expression decreased after 7 weeks of alcohol administration in another study (Angelogianni and Gianoulakis, 1993; Rasmussen et al., 2002). β-endorphin, a mediator of endogenous analgesia, the stress response, reward, cardiorespiratory regulation, and food intake, is also shown to modulate alcohol intake behavior (Lewis et al., 1987; Appleyard et al., 2003; Fields, 2004; Butler and Finn, 2009). Female transgenic mice with knockout of β-endorphin had significantly decreased alcohol consumption compared to control animals. Notably, stress exposure in β-endorphin knockout mice did not increase alcohol intake as it does in wild-type mice while showing no difference in baseline alcohol preference using two-bottle choice (Racz et al., 2008). In addition, many pharmacological studies demonstrate that antagonizing opioid receptors decreases alcohol intake, self-administration, and relapse in rodents (Altshuler et al., 1980; Volpicelli et al., 1986; Heyser et al., 1999). Opioid antagonists have also been shown to decrease drinking, craving, and relapse in AUD in humans as well (O’Malley et al., 1992, 2002; Volpicelli et al., 1992, 1997; Mason et al., 1994, 1999; Doty and de Wit, 1995). POMC neurons in the NTS also produce β-endorphin (Bronstein et al., 1992). These neurons project to regions in the brainstem associated with autonomic processing and pain control where they influence nociceptive processing and cardiorespiratory activity via β-endorphin release (Cerritelli et al., 2016). How NTS POMC neuronal activity affects alcohol-related behaviors has yet to be explored. The role that these neurons play in suppression of consumption along with their analgesic effects may be relevant for mediating alcohol intake and managing the negative reinforcing properties of alcohol withdrawal. POMC neurons in the arcuate nucleus of the hypothalamus and other central regions have been more extensively studied in the context of chronic alcohol exposure, resulting in alterations in β-endorphin, MSH and ACTH function (Navarro, 2017). There are minimal findings suggesting NTS POMC neurons and β-endorphin, MSH and ACTH are similarly changed by chronic alcohol exposures, however this should be investigated further.

## Neurotensin

Neurotensin is a neuropeptide that is expressed throughout the body including centrally, peripherally, and in the GI tract. Most notably, neurotensin was discovered to be present in limbic areas of the brain involved in reward such as the VTA, NAc and CeA (Uhl et al., 1979; Servonnet et al., 2017; Torruella-Suárez et al., 2020; Torruella-Suárez and McElligott, 2020). In the NAc, neurotensin has pre and postsynaptic effects on dopaminergic signaling (Kalivas et al., 1984). Thus, agonizing neurotensin receptors may be able to reduce alcohol intake by inhibiting alcohol induced dopamine release in the NAc.

In the CeA, alcohol consumption has been shown to increase *Fos* expression in neurotensin neurons. When these neurons are selectively deleted via cell-type specific viral manipulations in the CeA, alcohol consumed was significantly reduced. This reduction was not due to changes in overall fluid intake, motivation to intake rewarding substances, or aversion to alcohol (Torruella-Suárez et al., 2020). Thus, the neurotensin neurons in the CeA appears to be sufficient as a mechanism for targeted AUD therapies. Some neurotensin positive neurons are found in the DVC (Uhl et al., 1979; Triepel et al., 1984) as well as other autonomic regulatory regions in the medulla oblongata. The caudal NTS is also densely populated with neurotensin receptors (Higgins et al., 1984). While there are studies that look at neurotensin’s effects on drug seeking, reward and consumption behaviors in limbic areas of the brain, it is not known where neurotensin neurons in the NTS project to and what their functioning affects (Torruella-Suárez et al., 2020; Torruella-Suárez and McElligott, 2020). However, NTS neurotensin may also have similar roles in modulating alcohol consumption, potentially via projections to the CeA or other limbic regions. Such studies would be interesting to pursue in relation to AUD.

## Cholecystokinin

Cholecystokinin (CCK) is a peptide that is expressed throughout the GI tract and the central nervous system. It is also the most abundant neuropeptide encountered in the brain (Crawley and Corwin, 1994). Signaling by this peptide at CCK1 and CCK2 receptors has various physiological (Crawley and Corwin, 1994) consequences including stimulating gastric and pancreatic secretion, delaying gastric emptying as well as decreasing consumption through inhibiting orexigenic signaling in the hypothalamus (Chandra and Liddle, 2007). In addition to satiety, CCK has been shown to be involved in learning and memory, nociception, reward, and anxiety-related behaviors (Daugé and Léna, 1998; Crespi et al., 2000; Hadjiivanova et al., 2003; Rotzinger and Vaccarino, 2003; Keppel Hesselink, 2020). This neuropeptide modulates various transmitter systems in the brain including dopamine (Rotzinger et al., 2002), opioids (Hebb et al., 2005), glutamate (Nguyen et al., 2020), GABA (Freund and Katona, 2007), and serotonin (Grignaschi et al., 1993). Most notably, CCK is implicated in modulating the various positive and negative features of AUD (Ballaz et al., 2021).

The current studies with a focus on CCK neurons in the caudal NTS are centered around their roles in energy homeostasis. NTS CCK neurons are sensitive to satiation cues and their activation decreases feeding (D’Agostino et al., 2016; Roman et al., 2017). These neurons project to the PBN and PVN and activation of either set of pathways results in decreased food intake. The manner in which these different NTS CCK projections decrease food intake has been suggested to be opposite in valence. Activation of CCK neurons that project to the PBN is aversive while stimulation of CCK neurons that terminate in the PVN leads to pleasant satiation (Roman et al., 2016, 2017). Activation of NTS CCK neurons that terminate in the PBN also induces anxiety-like behaviors (Roman et al., 2016). Based on these initial findings, future studies examining NTS CCK neurons in relation to AUD may be warranted.

## Thyrotropin-Releasing Hormone

Thyrotropin-releasing hormone (TRH) is a neuropeptide that inhibits drinking and feeding behaviors (Vijayan and McCann, 1977; Morley and Levine, 1980). There are an abundance of TRH-immunopositive neurons in the DMV while a moderate amount are localized in the NTS (Fodor et al., 1994). Vagotomy attenuates the satiety effects of peripheral administration of TRH (Morley et al., 1982), suggesting the DVC may play a critical role in relaying TRH signaling to brain regions involved in controlling intake behaviors, but this has not been directly tested. TRH or its metabolite histidyl-proline diketopiperazine, may serve as a potential molecule for signaling satiation for alcohol, similar to GLP-1. An analog of TRH was shown to decrease alcohol preference in the alcohol preferring P rat line in a dose dependent manner while not affecting food intake (Rezvani et al., 1992; Mason et al., 1997). Overall, these findings suggest TRH as an additional DVC neuropeptide of potential interest in AUD research.

## Galanin

Galanin is a highly conserved neuropeptide that is found in the central nervous system as well as the peripheral nervous system and endocrine system (Vrontakis, 2002). It is thought to be a component in regulating nutrient intake (Fang et al., 2011) and has been implicated in cognitive functions such as learning and memory and affective states including anxiety (Wrenn and Crawley, 2001; Holmes et al., 2003; Barrera et al., 2006). Galanin has been shown to be highly co-localized with NE neurons in the pons and the LC as well as possibly participating in similar roles in stress in conjunction with NE (Melander et al., 1986; Holets et al., 1988; Hökfelt et al., 1998). There is immunoreactivity for galanin in the NTS, however, the functionality of these neurons and the location of their projections have not been examined (Maley, 1996).

Studies point to galanin acting as a modulator of various neurotransmitters through inhibition. Galanin tends to hyperpolarize neurons and can inhibit the release of NE as well as glutamate and serotonin (Pieribone et al., 1995; Xu et al., 1998; Kehr et al., 2002). Galanin may also have a role in alcohol consumption behaviors. Galanin injection in to the PVN increases dopamine release in the NAc (Rada et al., 1998). Galanin can increase alcohol intake when injected into the PVN. This increased alcohol intake then resulted in increased galanin release which implies that there may be a positive feedback loop between alcohol consumption and galanin release in the PVN (Lewis et al., 2005). Like other neuropeptides, galanin requires stronger levels of neural activation to be released when compared to neurotransmitters which makes activity-dependent modulation of signaling between NE and galanin likely (Barrera et al., 2006) and suggests that the galanin system may be a potential target for future AUD treatment development.

## Additional Neuropeptides

The NTS contains neurons that produce a number of other neuropeptides that have previously been investigated in relation to AUD, such as Met-enkephalin, Dynorphin, Substance P, Calcitonin Gene Related Peptide and Neuropeptide Y, but the specific role of NTS neurons that produce these peptides has essentially not been previously investigated. Such studies may inform on the overall mechanisms by which these neuropeptides may regulate alcohol intake behaviors and provide specific context to NTS function in AUD development.

## Clinical Relevance of Dorsal Vagal Complex Neurotransmitter and Peptide Systems in Alcohol Use Disorder

Although the role of the DVC in AUD is not well characterized, a number of studies suggest that the DVC may be a critical area of interest for future AUD therapies. Since the DVC is a primary input region of the vagus and other peripheral nerves and regulates autonomic output, the DVC and its various neurotransmitter systems can be examined and/or modulated, either directly or indirectly, in clinical and preclinical studies. This allows for a number of future translational studies that can impact AUD research.

Heartrate variability (HRV), an indirect measure of parasympathetic (vagal) tone to the heart, has been established as a valuable biomarker of autonomic health. Recent evidence shows that AUD patients have altered HRV suggesting that these patients have reduced parasympathetic activity and autonomic dysfunction due to alcohol misuse (Yuksel et al., 2016; Cheng et al., 2019). HRV could also be used for informing AUD prognosis since its measurements are associated with drug craving, risky drinking behaviors and relapse (Milivojevic and Sinha, 2018; Ralevski et al., 2019; Hwang et al., 2021). Thus, the monitoring and modulation of DVC pathways and vagal activity may be a critical and under-explored aspect of AUD research. HRV could serve as an innovative diagnostic tool to detect disruption in central neuronal networks by AUD and other substance use disorders.

Of clinical relevance to the DVC gut-brain peptides discussed above, there has been a recent surge of bariatric surgical procedures (∼200,000 per year) as they represent one of the most effective interventions to achieve significant, long-term weight loss to treat medical comorbidities of obesity (Nguyen and Varela, 2017). However, one common procedure, the Roux-en-Y gastric bypass surgery (RYGB) can lead to increased alcohol use and AUD in humans, even in those individuals without a history of significant alcohol use, with similar findings in rodent models (Hajnal et al., 2012; Thanos et al., 2012; Davis et al., 2013; Polston et al., 2013; Blackburn et al., 2017; King et al., 2017). While the exact underlying mechanism remains unknown, pre-clinical studies of RYGB points to neuronal plasticity occurring in the DVC following removal of gastric vagal afferents (Browning et al., 2013; Browning and Hajnal, 2014; Ballsmider et al., 2015; Minaya et al., 2019). One recent preclinical study focused on damage to the gastric branches of the vagus nerve that is unavoidable in the RYGB procedure and found gastric vagal de-afferentation is sufficient to increase alcohol intake in both obese and lean rats (Orellana et al., 2021b), suggesting vagal afferent dysfunction and resulting alterations in DVC activity may play a critical role in AUD development.

Another potential mechanism is altered ghrelin signaling following RYGB. This notion has been further supported by differential AUD outcomes seen following RYGB and vertical sleeve gastrectomy, the two most common weight loss surgeries that may differentially alter ghrelin production as well as involving vagal and central ghrelin receptor signaling in development of postoperative AUD (Orellana et al., 2018, 2019, 2021a). Lastly, emerging evidence suggests that changes in gut microbiota diversity and composition following bariatric surgery may influence AUD outcomes (Martin et al., 2021). Whether such changes are related to altered diet and/or reduced obesity (Sen et al., 2017; Vaughn et al., 2017; Stephens et al., 2018; Minaya et al., 2020), and the extent of which vagus and DVC play role in mediating gut microbiota-related effects in general (Kim et al., 2020), and in AUD in particular (Fuenzalida et al., 2021; Leclercq et al., 2021; Russell et al., 2021), remain high priority questions for translational research thus warrant future mechanistic studies.

Overall, the above studies suggest that disruption to normal vagus nerve signaling, and related disruptions to DVC function, may be a critical yet under-examined factor driving both AUD development and relapse. Corollary to this, vagus nerve stimulation (VNS) as a way to counteract disrupted vagus nerve signaling may hold promise as a novel therapeutic approach in AUD. VNS is already approved as an adjunctive therapy for patients with epilepsy and depression that are resistant to pharmacological treatment, however the mechanisms involved in these effects remains elusive (Senova et al., 2019; Toffa et al., 2020). Preclinical studies with VNS reveal that activating afferent vagal sensory neurons via electrode stimulation alters central neuronal signaling which may help rectify circuits regulating heroin and cocaine intake and seeking (Liu et al., 2011; Childs et al., 2017). In rodents, VNS led to increases in extracellular concentrations of NE and serotonin in the cortex and hippocampus (Roosevelt et al., 2006; Follesa et al., 2007; Manta et al., 2009) although other neurotransmitter and neuropeptide systems in the DVC and their projections to forebrain and midbrain regions are also likely to be involved. Further examination of the effects of VNS in preclinical models of AUD is needed to elucidate the modality of its influence on AUD pathology.

The evidence from recent clinical studies with VNS in substance use disorders are promising. Transcutaneous auricular VNS (taVNS), a non-invasive form of vagal stimulation targeting the auricular branch of the vagus nerve around the ear, has been tested as an adjuvant to oral morphine therapy in infants with neonatal opioid withdrawal syndrome. When taVNS is delivered before morphine delivery, the length of morphine treatment was significantly reduced (Wang et al., 2021). taVNS has also been shown to reduce self-reported anxiety and depression symptoms as well as improve sleep quality in AUD patients after withdrawal (Follesa et al., 2007). Currently there is a clinical trial examining the effects of taVNS on alcohol craving and drinking behaviors, which further examines activation of brain regions associated with craving and functional connectivity of neural networks related to addiction^1^. The concurrent development of VNS therapies in clinical practice along with VNS research in translational addiction models will help to improve understanding of the significance of brain-body communication via vagal/DVC circuitry in the development and treatment of AUD. Overall, these studies suggest that further examination of vagus nerve and DVC function in AUD is a promising avenue for discovery of future therapeutic options.

## Conclusion

The DVC is an intricate brainstem region with a rich variety of neurotransmitters and neuropeptides. These signaling molecules help to relay endocrine and sensory information from the body to the brain. Visceral vagal afferents in the GI tract, heart, liver, and lungs are affected by alcohol consumption (Siegmund et al., 2003). In addition, neural activity in vagal nuclei in the brainstem are also independently affected by acute and chronic alcohol exposure (Covarrubias et al., 2005; Aimino et al., 2018). In this review, we discussed how the DVC projects to and modulates systems such as the homeostatic control system and cortisol system in the hypothalamus as well as the reward system and stress system in limbic regions. These systems are also disrupted by alcohol consumption and withdrawal. The numerous functions of DVC neuropeptides and neurotransmitters within different systems needs to be kept in mind when interpreting the effects of these molecules as well as their roles in modulating alcohol-related behaviors. Modulating endogenous activity of these various systems with gut brain peptide analogs and vagal activity manipulations provide promising avenues for developing effective clinical therapies for AUD. Further study of DVC neurocircuitry, neurotransmitters and neuropeptides will be useful in understanding how this region influences systems recruited during chronic alcohol consumption and ultimately will be integral for developing targeted AUD treatments.

## Author Contributions

BK and YS contributed to the conception, design, and drafting of the manuscript. BK, YS, AH, KB, and AA revised and edited the manuscript. All authors contributed to the article and approved the submitted version.

## Conflict of Interest

The authors declare that the research was conducted in the absence of any commercial or financial relationships that could be construed as a potential conflict of interest.

## Publisher’s Note

All claims expressed in this article are solely those of the authors and do not necessarily represent those of their affiliated organizations, or those of the publisher, the editors and the reviewers. Any product that may be evaluated in this article, or claim that may be made by its manufacturer, is not guaranteed or endorsed by the publisher.

## Footnotes

1
https://clinicaltrials.gov/ct2/show/NCT04106739
